# Cytochrome P450 2D6 (CYP2D6) and glucose-6-phosphate dehydrogenase (G6PD) genetic variations in Thai vivax malaria patients: Implications for 8-aminoquinoline radical cure

**DOI:** 10.1371/journal.pntd.0010986

**Published:** 2022-12-12

**Authors:** Kamonwan Chamchoy, Sirapapha Sudsumrit, Thanyapit Thita, Srivicha Krudsood, Rapatbhorn Patrapuvich, Usa Boonyuen

**Affiliations:** 1 Princess Srisavangavadhana College of Medicine, Chulabhorn Royal Academy, Bangkok, Thailand; 2 Department of Molecular Tropical Medicine and Genetics, Faculty of Tropical Medicine, Mahidol University, Bangkok, Thailand; 3 Drug Research Unit for Malaria (DRUM), Faculty of Tropical Medicine, Mahidol University, Bangkok, Thailand; 4 Department of Tropical Hygiene, Faculty of Tropical Medicine, Mahidol University, Bangkok, Thailand; Kenya Agricultural and Livestock Research Organization, KENYA

## Abstract

**Background:**

Primaquine and tafenoquine are the only licensed drugs that effectively kill the hypnozoite stage and are used to prevent *Plasmodium vivax* malaria relapse. However, both primaquine and tafenoquine can cause acute hemolysis in glucose-6-phosphate dehydrogenase (G6PD)-deficient people with varying degrees of severity depending on G6PD variants. Additionally, primaquine efficacy against malaria parasites was decreased in individuals with impaired cytochrome P450 2D6 (CYP2D6) activity due to genetic polymorphisms. This study aimed to characterize *G6PD* and *CYP2D6* genetic variations in vivax malaria patients from Yala province, a malaria-endemic area along the Thai–Malaysian border, and determine the biochemical properties of identified G6PD variants.

**Methodology/Principle findings:**

Multiplexed high-resolution melting assay and DNA sequencing detected five G6PD variants, including G6PD Kaiping, G6PD Vanua Lava, G6PD Coimbra, G6PD Mahidol, and G6PD Kerala-Kalyan. Biochemical and structural characterization revealed that G6PD Coimbra markedly reduced catalytic activity and structural stability, indicating a high susceptibility to drug-induced hemolysis. While Kerala-Kalyan had minor effects, it is possible to develop mild adverse effects when receiving radical treatment. *CYP2D6* genotyping was performed using long-range PCR and DNA sequencing, and the phenotypes were predicted using the combination of allelic variants. Decreased and no-function alleles were detected at frequencies of 53.4% and 14.2%, respectively. The most common alleles were CYP2D6*36+*10 (25.6%), *10 (23.9%), and *1 (22.2%). Additionally, 51.1% of the intermediate metabolizers showed CYP2D6*10/*36+*10 as the predominant genotype (15.9%).

**Conclusions/Significance:**

Our findings provide insights about genetic variations of *G6PD* and *CYP2D6* in 88 vivax malaria patients from Yala, which may influence the safety and effectiveness of radical treatment. Optimization of 8-aminoquinoline administration may be required for safe and effective treatment in the studied population, which could be a significant challenge in achieving the goal of eliminating malaria.

## Author summary

Use of 8-aminoquinolines, which are an anti-relapse therapy for *P*. *vivax*, is limited because of the risk of life-threatening hemolysis in individuals with G6PD deficiency. Impaired CYP2D6 affecting primaquine biotransformation is another challenge for effective radical cure of vivax malaria. *G6PD* and *CYP2D6* are highly polymorphic genes that vary geographically and ethnically. Therefore, information on *G6PD* and *CYP2D6* genetic variations should be assessed. Class B G6PD variants were discovered in Yala province, suggesting that anti-relapse medications may result in a moderate to severe hemolytic crisis in the absence of screening for G6PD deficiency. Up to 51.1% of vivax patients in this region were intermediate metabolizers with decreased CYP2D6 activity, putting them at risk of therapeutic failure. On the basis of the population’s genetic profiles, we recommend ensuring robust screening for G6PD deficiency and monitoring effectiveness of current 8-aminoquinoline regimens.

## Introduction

*Plasmodium vivax* remains a public health problem, especially in Southeast Asia and America where approximately 50% and 70% of malaria cases are caused by *P*. *vivax*, respectively [1]. A recent study showed that the number of *P*. *vivax* infections is increasing across malaria-endemic regions in Africa [2]. The burden of *P*. *vivax* is caused by the challenges in controlling the disease. *P*. *vivax* is difficult to treat because of its dormant liver stage (hypnozoites) that can reactivate weeks to months after the initial infection, resulting in relapse and ongoing transmission. Hypnozoite reactivation is also associated with significant morbidity and mortality in vivax infection. Frequent relapses cause severe anemia, which has negative clinical impacts, particularly in young children and pregnant women [3,4]. An effective treatment that prevents relapse in people infected with *P*. *vivax* is required to achieve the malaria elimination goal. Primaquine and tafenoquine are the only licensed 8-aminoquinolines used for radical cure of *P*. *vivax*. To prevent relapse, a 14-day course of primaquine at a dose of 0.25 mg/kg/day or 15 mg (standard dose) is recommended in most regions, while 0.5/kg/day or 30 mg (high dose) is recommended in East Asia and Oceania. Tafenoquine is prescribed as a single dose of 300 mg [5–7]. Unfortunately, the use of primaquine and tafenoquine is complicated by genetic variations of glucose-6-phosphate dehydrogenase (G6PD). Additionally, efficacy of primaquine is determined by polymorphisms in cytochrome P450 2D6 (CYP2D6).

G6PD is an enzyme that catalyzes the formation of reduced nicotinamide adenine dinucleotide phosphate (NADPH), a reducing agent required to protect red blood cells from the oxidative stress. Additionally, primaquine and tafenoquine can cause dose-dependent acute hemolytic anemia in G6PD-deficient individuals [6,8]. G6PD deficiency, an X-linked recessive disorder, affects approximately 500 million people worldwide. It is less common and highly heterogeneous in Americans, depending on ethnicity and genetic heritage. The prevalence of G6PD deficiency is high in malaria-endemic areas, and it is presumed to be the result of natural selection by survival advantage against malaria [9,10]. The majority of G6PD-deficient people are Africans and Asians [9]. The World Health Organization (WHO) recommends G6PD testing before prescribing 8-aminoquinolines for radical cure. Malaria patients with <30% of normal G6PD activity can receive primaquine with a dose adjustment (45 mg weekly dose for 8 weeks) under medical supervision [11], and tafenoquine is only prescribed to those with more than 70% G6PD activity [12]. Clinical manifestations of drug-induced hemolysis vary from self-limiting to severe hemolysis that cannot be compensated and is life-threatening, depending on the genetic variant in the *G6PD* gene. Over 200 G6PD variants have been identified to date [9]. G6PD variants were previously classified into five classes, ranging from Class I variants with severe enzyme deficiency and chronic non-spherocytic hemolytic anemia (CNSHA) to Class V variants with increased enzyme activity and no clinical consequence [9,13]. The majority of identified G6PD variants are Class II and Class III, with the other variants having low frequencies or being clinically insignificant. However, many studies have reported that the severity of hemolysis and neonatal jaundice in Class II and Class III variants overlaps. Therefore, the WHO revised the classification in early 2022, dividing G6PD genetic variants into four classes. Class A are variants with median G6PD activity less than 20% and are linked to CNSHA. Class B are variants with median G6PD activity less than 45% and are susceptible to acute hemolysis when exposed to triggers. Class C are variants with median G6PD activity ranging from 60% to 150% and no clinical outcomes. Variants with any G6PD activity and evidence for hemolysis remains unclear are classified as Class U [14].

CYP2D6 is a key enzyme involved in the metabolism of a large number of commonly prescribed drugs. Primaquine is a prodrug requiring conversion by the CYP2D6 enzyme to be effective against malaria parasites, while the evidence for tafenoquine remains unclear. Therefore, the high polymorphic nature of *CYP2D6*, which affects its catalytic efficiency, makes using primaquine challenging. To date, over 140 allelic variants (*) of CYP2D6 have been identified, including single nucleotide polymorphisms, insertion/deletion, copy number variations, and hybridization with the *CYP2D7* pseudogene (https://www.pharmvar.org/gene/CYP2D6). These alleles can be classified as no function, decreased function, normal function, and increased function. The combination of alleles in an individual results in four phenotypic groups, including normal metabolizer (NM), poor metabolizer (PM, no enzyme activity), intermediate metabolizer (IM, decreased enzyme activity), and ultra-rapid metabolizer (UM, increased enzyme activity) [15,16]. The association between impaired CYPD26 and primaquine failure in treatment of vivax malaria patients was demonstrated in several studies. Clinical studies revealed that relapse was predominantly found in patients who were poor or intermediate metabolizers [17–20]. Multiple episodes of *P*. *vivax* relapse were reported in a patient who was an intermediate metabolizer with less than 50% CYP2D6 activity and treated with high-dose primaquine (30 mg daily for 14 days) to prevent malaria recurrence [21]. Taken together, genetic variations in CYP2D6 should be considered when administering primaquine in *P*. *vivax* patients.

In Thailand, the prevalence of G6PD deficiency ranges between 5% and 34%, and more than 20 G6PD variants have been identified. A high frequency of G6PD mutations was found in the malaria-endemic areas along the Thai–Myanmar border, and G6PD Mahidol (487 G>A) was the most common variant in this area [22–25], while G6PD Viangchan (871 G>A) was commonly found in central and southern regions [26–32]. For CYP2D6, CYP2D6*10, which causes a reduction in enzyme activity, is common in the Thai population, accounting for approximately 50% of all allelic variants [33–36]. Between 2019 and 2020, Thailand reported 9,375 malaria cases. There were 2,273 malaria cases in Yala province, accounting for 24% of all cases. Among them, 91% of malaria cases were identified as *P*. *vivax* [37]. This highlights the importance of 8-aminoquinolines treatment in overcoming the *P*. *vivax* burden in the area. Therefore, the profile of *G6PD* and *CYP2D6* polymorphisms in this population should be characterized in order to provide information about the safety and efficacy of using 8-aminoquinolines in the treatment of vivax malaria. In the present study, the genetic variations of *G6PD* and *CYP2D6* were determined in vivax patients from Yala province. Furthermore, the biochemical and structural properties of identified G6PD variants were characterized. The results will be beneficial for decision-making and effective management of vivax malaria patients in the studied population.

## Materials and methods

### Ethics statement

The study was approved by the Human Ethics Committee of the Faculty of Tropical Medicine, Mahidol University (approval number MUTM 2021-075-01) and the Chulabhorn Research Institute (approval number 087/2563). The patients/participants provided their written informed consent to participate in the study.

### Sample collection and processing

This retrospective study was carried out using venous blood samples collected in Yala province during 2019 to 2020. Blood samples were collected in ethylenediaminetetraacetic acid (EDTA) and *Plasmodium* infection was diagnosed by microscopic analysis of Giemsa-stained thin blood smears. All malaria-positive samples were enrolled and transported on dry ice to the laboratory. Identification of the *Plasmodium* species was performed using polymerase chain reaction (PCR)-based protocols [38]. Blood samples were aliquoted and kept at -20°C until use. In this study, a total of 88 blood samples were selected according to the following criteria; i) positive for *P*. *vivax* and ii) obtained before anti-malarial treatment. Genomic DNA (gDNA) was extracted using the QIAamp DNA Blood Mini Kits (QIAGEN, Hilden, Germany), following the manufacturer’s instructions. 500 μL blood sample was used and eluted into a final volume of 100 μL. DNA concentration was measured using a NanoDrop 2000 spectrophotometer (Thermo Fisher Scientific, Waltham, MA, USA).

### G6PD genotyping using multiplexed high-resolution melting (HRM) assays

Multiplexed high-resolution melting (HRM) assays were used to detect 8 G6PD variants common in Thailand and Southeast Asia, following our previous study [25]. A total volume of 12.5 μL was used for the multiplexed HRM assays, which included 6.25 μL of 1× HRM Type-It mix (QIAGEN), different concentrations of each primer, molecular-grade water, and 2.5 μL of the gDNA template. PCR amplification and melting curve analysis were carried out with the Rotor-Gene Q (QIAGEN) under the following conditions: 1 cycle at 95°C for 5 min, followed by 30 cycles at 95°C for 10 sec, 63°C for 30 sec, and 72°C for 10 sec. After that, HRM analysis was carried out by melting from 75 to 90°C and taking readings at every 0.1°C step with 2 sec of stabilization. Every run included both positive (gDNA with known mutations, confirmed by DNA sequencing) and negative controls (gDNA of G6PD wild-type (WT), confirmed by DNA sequencing). In addition, *β-actin* amplification was included as an internal control to ensure the integrity of the gDNA. The Rotor-Gene Q software was used to analyze the data.

DNA sequencing was performed to confirm G6PD status. The *G6PD* gene was amplified using previously described conditions [25]. PCR products were subjected to purification and sequenced (1st BASE, Apical Scientific, Malaysia).

### Biochemical and structural characterization of G6PD variants

#### Recombinant construction and expression of G6PD variants

G6PD Mahidol and G6PD Kaiping mutations were identified using HRM assays, whereas DNA sequencing revealed three additional G6PD mutations: G6PD Vanua Lava, G6PD Coimbra, and G6PD Kerala-Kalyan. While G6PD Vanua Lava has been thoroughly characterized, there is no previous information on the biochemical properties of G6PD Coimbra and G6PD Kerala-Kalyan [39]. Therefore, these two variants were investigated in terms of biochemical and structural properties to understand the molecular mechanisms underlying enzyme deficiency. G6PD mutations were created by site-directed mutagenesis using pET28a-G6PD WT as a template and the presence of desired mutations was confirmed by DNA sequencing. Primers used for site-directed mutation are listed in S1 Table. The PCR conditions for site-directed mutagenesis were previously described [40]. G6PD protein expression was achieved using *E*. *coli* BL21 (DE3), which were cultured at 37°C with 250 rpm shaking in the presence of 50 μg/mL kanamycin until the OD_600_ reached 1 and G6PD expression was induced using 1 mM IPTG. Cells were then cultured for 20 h at 20°C with 200 rpm shaking before being harvested by centrifugation. Protein purification was carried out using immobilized metal affinity chromatography in accordance with the previously described protocols [40]. Protein purity was visualized with sodium dodecyl sulfate-polyacrylamide gel electrophoresis (SDS-PAGE) and the Bradford assay was used to determine protein concentration.

#### Determination of steady-state kinetic parameters

Steady state kinetic parameters were determined to assess the effect of mutations on catalytic activity of G6PD variants. The standard reaction contained 20 mM Tris-HCl pH 8.0, 10 mM MgCl_2_, 500 μM G6P and 100 μM NADP^+^. The enzymatic reaction was monitored by following the formation of NADPH at 340 nm using UV-VIS spectrophotometer (Shimadzu, Kyoto, Japan). To determine the *K*_m_ for G6P, the concentration of NADP^+^ was fixed at 100 μM while varying concentrations of G6P from 2.5 to 1000 μM and to determine the *K*_m_ for NADP^+^, the concentration of G6P was fixed at 500 μM while varying concentrations of NADP^+^ from 1 to 200 μM.

#### Determination of secondary structure

The secondary structure was analyzed using circular dichroism (CD) to determine the effect of mutations on secondary structure of G6PD variants. Far UV-CD spectra of the G6PD variants (0.1 mg/mL) were recorded in a 1 mm path-length quartz cuvette at 25°C using a Jasco spectrometer, model J-815, equipped with a Peltier temperature control system. The CD spectra were collected at a scan rate of 50 nm/min over a wavelength range of 190–260 nm. Five scans were averaged for each sample, and the results of the buffer scans were subtracted.

#### Determination of structural stability

The effect of mutations on structural stability was assessed by determining the susceptibility of G6PD variants to guanidine hydrochloride (Gdn-HCl) treatment and trypsin digestion, and thermal stability analysis. To determine susceptibility of G6PD variants to trypsin digestion, the protein was treated with trypsin (0.5 mg/mL) for 5 min at 25°C in the presence of various concentrations of NADP^+^ (0, 10 and 100 μM). The residual enzyme activity was measured and expressed as a percentage of the activity of the same enzyme incubated without trypsin.

To investigate structural stability of G6PD variants upon chemical denaturation, the protein was treated with different concentrations of Gdn-HCl (0 to 0.5 M) in the presence of various concentrations of NADP^+^ (0, 10 and 100 μM) at 37°C for 2 h. The residual enzyme activity was measured and expressed as a percentage of the activity of the same enzyme incubated without Gdn-HCl.

Finally, thermal stability analysis was carried out in a 20 μL reaction, containing protein at a concentration of 0.25 mg/mL mixed with 5 × SYPRO Orange Protein Gel Stain (Thermo Fisher Scientific, San Jose, CA, USA). The reaction mixtures were heated in a Light- Cycler 480 real-time PCR machine (Roche, Mannheim, Germany) at temperatures ranging from 20 to 80°C, with excitation and emission wavelengths of 465 and 580 nm, respectively. Furthermore, the effect of NADP^+^ was investigated by incubating the protein with various concentrations of NADP^+^ (0, 10 and 100 μM). The melting temperature (*T*_*m*_) of each G6PD variant was calculated and defined as the temperature at which half of the protein unfolded.

### CYP2D6 genotyping and phenotype prediction

**Fig 1:**
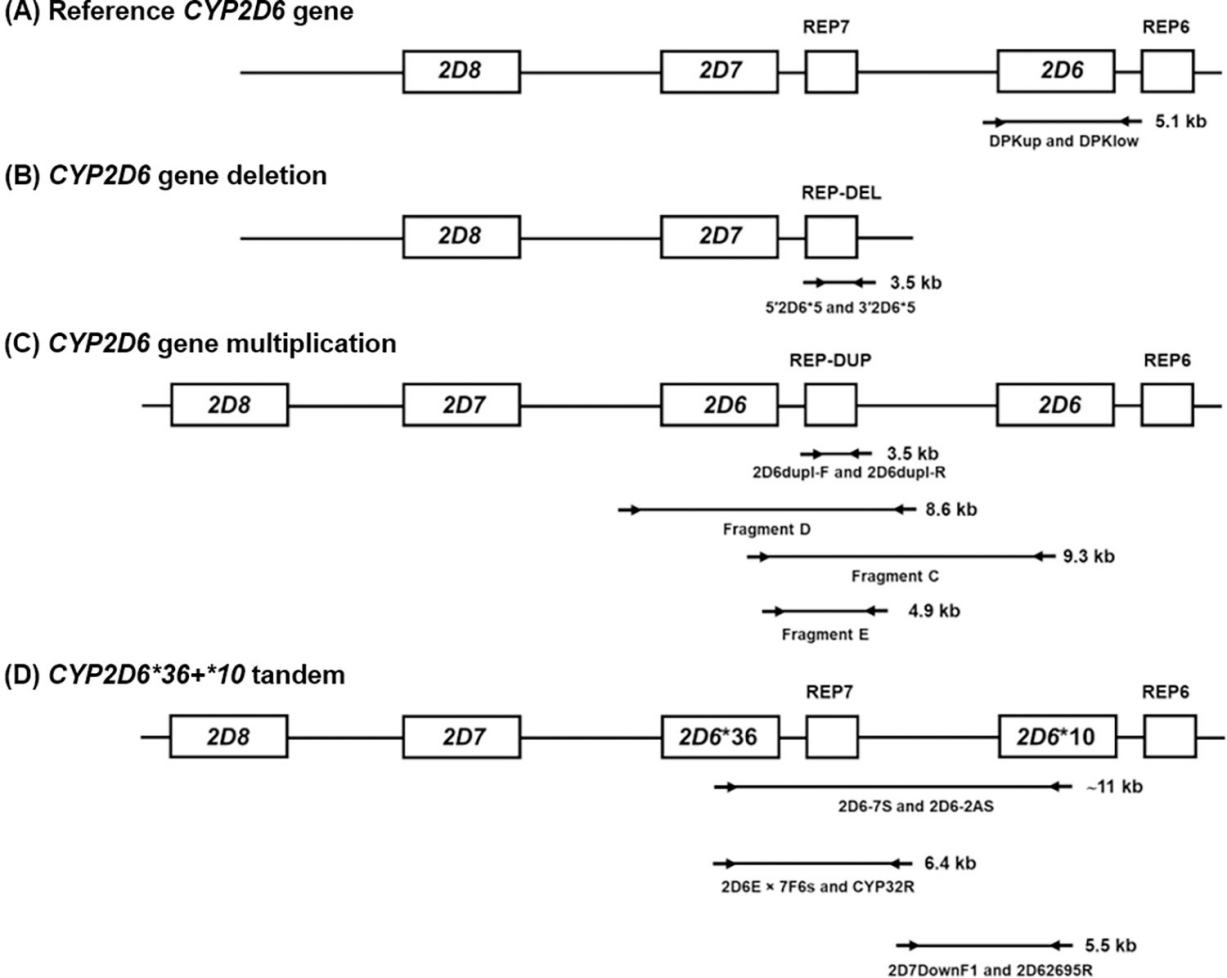
An overview of the *CYP2D6* gene structure and the primers used to detect deletion, multiplication and CYP2D6*36+*10 tandem.

The human *CYP2D6* gene is known to be highly polymorphic, with varying levels of enzyme activity. The *CYP2D6* gene was amplified and sequenced to identify single nucleotide polymorphisms (SNPs) and copy number variation (CNV) among 88 vivax malaria positive samples, using primers listed in S2 Table. **Fig 1** depicts the gene structure and primers used to detect the CYP2D6 genotype.

### Detection of single nucleotide polymorphisms (SNPs)

To amplify the entire *CYP2D6* gene, the first PCR reaction was set up in a final volume of 50 μL, containing 1x PrimeSTAR GXL buffer, 200 μM of each dNTP, 0.25 μM of each primer, 20 ng gDNA and 1.25 U of PrimeSTAR GXL DNA polymerase (Takara Bio, San Jose, CA, USA). The thermal cycling profile was as follows: initial denaturation at 95°C for 5 min; 30 cycles of denaturation at 98°C for 10 s and annealing at 72°C for 5 min; then followed by final extension at 72°C for 7 min. PCR products (expected size 5.1 kb) were visualized by agarose gel electrophoresis. Three subregions of *CYP2D6* gene were subsequently amplified using the nested PCR primer sets (subregions 1–3). The second PCR was performed in a final volume of 50 μL, containing 1x Taq buffer with (NH_4_)_2_SO_4_, 2.5 mM MgCl_2_, 200 μM of each dNTP, 0.25 μM of each primer, 1.5 μL of first round PCR products and 1.25 U of Taq DNA polymerase (Thermo Fisher Scientific). The thermal cycling profile was as follows: initial denaturation at 95°C for 3 min; 35 cycles of denaturation at 95°C for 30 s, annealing at 60°C for 30 s, and extension at 72°C for 2 min; followed by final extension at 72°C for 5 min. PCR products were purified and sequenced (1st BASE, Apical Scientific, Malaysia).

#### Detection of gene deletion and multiplication

A PCR reaction was set up in a final volume of 50 μL, containing 1× PrimeSTAR GXL buffer, 200 μM of each dNTP, 0.25 μM of each primer, 20 ng gDNA and 1.25 U of PrimeSTAR GXL DNA polymerase. The thermal cycling profile was as follows: initial denaturation at 95°C for 5 min; 30 cycles of denaturation at 98°C for 10 s and annealing at 72°C for 5 min, followed by final extension at 72°C for 7 min. PCR products (expected size 3.5 kb) were visualized using agarose gel electrophoresis. Additionally, to further confirm the presence of two or more *CYP2D6* gene copies, three additional fragments were amplified (fragments C, D, and E). PCR was performed in a final volume of 25 μL using a 1× KAPA Long Range Hot Start PCR Kit, 200 μM of each dNTP, 0.25 μM of each primer, 20 ng gDNA, and 0.625 U of KAPA Long Range Hot Start DNA polymerase (Sigma Aldrich, St. Louis, MO, USA). The thermal cycling profile was as follows: initial denaturation at 95°C for 5 min; 35 cycles of denaturation at 95°C for 20 s, annealing at 66°C for 10 min, followed by final extension at 72°C for 10 min. PCR products were visualized using agarose gel electrophoresis.

#### Detection of CYP2D6*36 + *10 tandem

Long range PCR was used to detect the presence of CYP2D6*36 + *10 tandem-type, which carries a CYP2D6*36 gene upstream of a CYP2D6*10. The first round PCR was performed in a final volume of 25 μL, using 1× KAPA Long Range Hot Start PCR Kit, 200 μM of each dNTP, 0.25 μM of each primer, 1.5 μL of first round PCR products, and 0.625 U of KAPA Long Range Hot Start DNA polymerase. The thermal cycling profile was as follows: initial denaturation at 95°C for 5 min; 30 cycles of denaturation at 95°C for 30 s, and annealing at 72°C for 11 min, followed by final extension at 72°C for 10 min. PCR products were visualized using agarose gel electrophoresis. Thereafter, two subregions were amplified using nested PCR primer sets. The second PCR was performed in a final volume of 25 μL, using a 1× KAPA Long Range Hot Start PCR Kit, 200 μM of each dNTP, 0.25 μM of each primer, 20 ng gDNA, and 0.625 U of KAPA Long Range Hot Start DNA polymerase. The thermal cycling profile was as follows: initial denaturation at 95°C for 5 min; 30 cycles of denaturation at 95°C for 30 s, annealing at 65°C for 30 s, and extension at 72°C for 6 min, followed by final extension at 72°C for 7 min. PCR products were visualized using gel electrophoresis.

#### Designation of *CYP2D6* alleles and phenotype prediction

Star (*) allele designations were performed according to the *CYP2D6* allele nomenclature database described by the Pharmacogene Variation (PharmVar) Consortium (https://www.pharmvar.org/gene/CYP2D6). Each star allele was assigned functional status with either normal, decreased, no, uncertain, or unknown function as defined by the Clinical Pharmacogenomics Implementation Consortium (CPIC). Subsequently, activity score (AS) of the diplotype was calculated and translated into phenotype. Individuals with AS of 0 are poor metabolizers, AS of 0.25 to 1 are intermediate metabolizers, AS of 1.25 to 2.25 are normal metabolizers, AS greater than 2.25 are ultra-rapid metabolizers. While those carrying uncertain/unknown alleles in the genotype are defined as indeterminate (https://www.pharmgkb.org/page/cyp2d6RefMaterials) [15,16].

## Results

### Genotypic analysis of G6PD deficiency

Using multiplexed HRM assays, G6PD genotyping revealed two samples, from one male and one female, carrying G6PD Kaiping (G1388A, Arg463His) and G6PD Mahidol (G487A, Gly163Ser), respectively. Hemizygous male carried the Kaiping mutation whereas heterozygous female carried the Mahidol mutation. Direct DNA sequencing revealed the presence of the following three other mutations in the studied samples: G6PD Coimbra (C592T, Arg198Cys), G6PD Kerala-Kalyan (G949A, Glu317Lys), and G6PD Vanua Lava (T383C, Leu128Pro). G6PD Kerala-Kalyan was detected in a man, while G6PD Coimbra and G6PD Vanua Lava were identified in heterozygous females (Table 1).

**Table 1:** Genotypic analysis of G6PD deficiency.

Sex	Genotype	Zygosity	Classification ^a^
Male	Kaiping	Hemizygous	B
Male	Kerala-Kalyan	Hemizygous	B
Male	C1311T and IVS XI T93C	Hemizygous	n/a
Male	C1311T and IVS XI T93C	Hemizygous	n/a
Female	Vanua Lava	Heterozygous	B
Female	Coimbra	Heterozygous	B
Female	Mahidol	Heterozygous	B
Female	C1311T and IVS XI T93C	Heterozygous	n/a
Female	C1311T and IVS XI T93C	Heterozygous	n/a

^a^ Revised classification by WHO (2022), Class B variants have median G6PD activity <45% with acute hemolysis upon exposure to triggers. n/a, no G6PD classification is assigned.

## Biochemical and structural characterization of G6PD variants

Kinetic properties of G6PD variants are shown in Table 2. The Coimbra mutation had a significant impact on catalytic activity, resulting in an 11-fold decrease, while the Kerala-Kalyan mutation only had a minor effect, causing a two-fold decrease. Both mutations had no effect on the binding affinity toward NADP^+^ substrate, but increased binding affinity toward G6P substrate.

**Table 2:** Kinetic parameters of G6PD variants.

Construct	Amino acid change	*k*_cat_ (s^–1^)	*K*_m_ G6P (μM)	*K*_m_ NADP^+^ (μM)
WT	-	329 ± 15	45 ± 6	9 ± 1
Coimbra	Arg198Cys	29 ± 4	18 ± 3	10 ± 3
Kerala-Kalyan	Glu317Lys	143 ± 8	13 ± 2	7 ± 2

**Fig 2:**
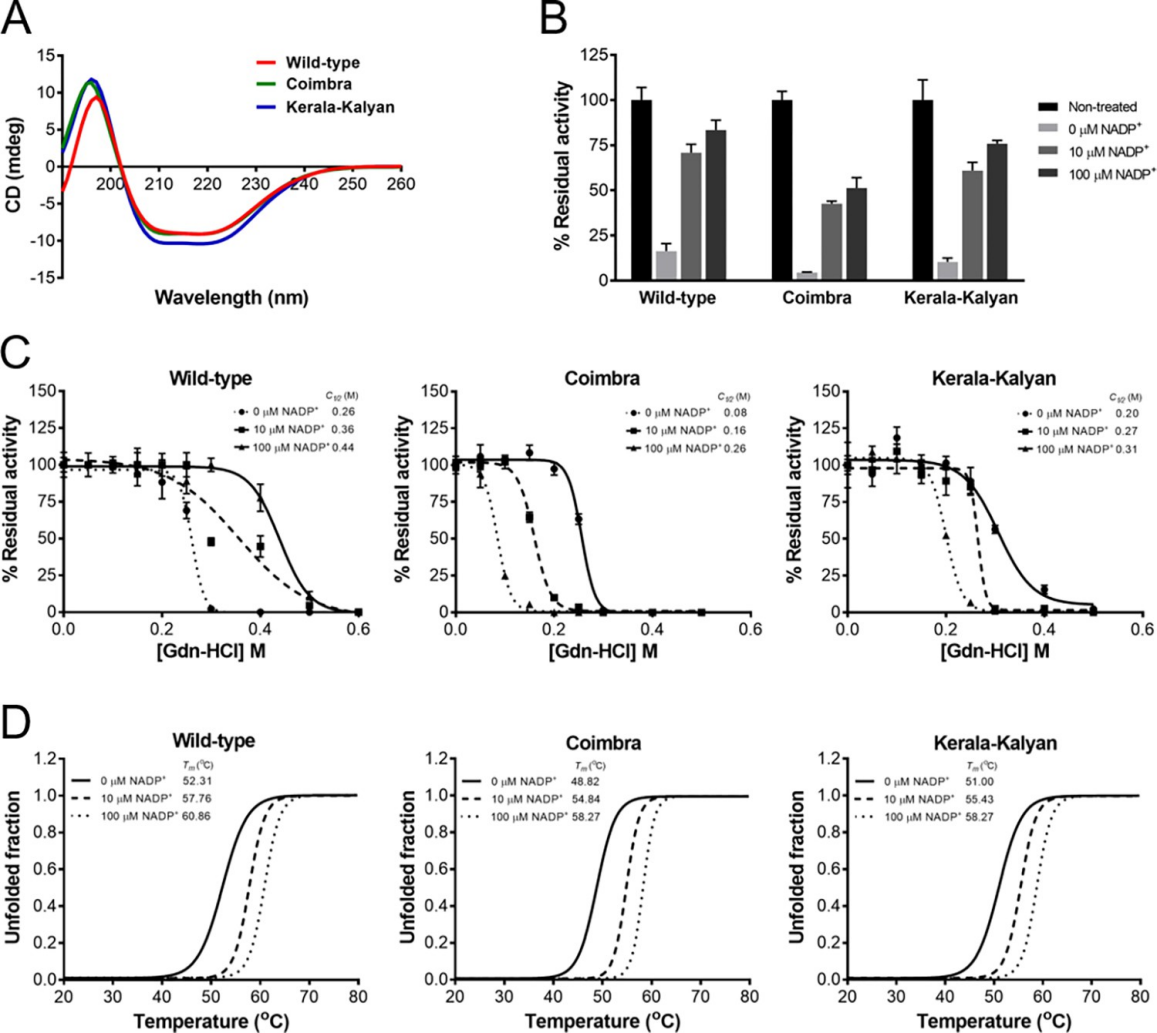
Biochemical and structural characterization of recombinant G6PD variants. (A) Far-UV spectra were recorded over a wavelength range of 190 to 260 nm. (B) Susceptibility to trypsin digestion in the presence of various concentrations of NADP^+^. (C) Stability analysis upon Gdn-HCl treatment in the presence of various concentrations of NADP^+^. (D) Thermal stability analysis in the presence of various concentrations of NADP^+^. Error bars represent the mean ± SD of triplicate measurements.

The effects of mutations on structural properties of G6PD variants were assessed. First, to investigate the effects of mutations on secondary structure of G6PD Coimbra and G6PD Kerala-Kalyan, CD spectra of these two variants were recorded and compared with that of the G6PD WT (Fig 2A). The three-dimensional structure of the human G6PD protein is composed of two domains, which are the β+α domain and a coenzyme binding domain with a classic β-α-β dinucleotide-binding fold [41]. Here, CD spectra revealed that all G6PD proteins exhibited a similar absorption pattern, with maximum negative absorbance at 208 and 222 nm, which are α-helical protein characteristics. G6PD WT and G6PD Coimbra had nearly identical negative absorption intensities, whereas G6PD Kerala-Kalyan had a higher intensity, indicating a change in secondary structure rigidity or flexibility. However, G6PD Coimbra and G6PD Kerala-Kalyan showed a slight alteration in β absorption (a positive band between 195 and 200 nm), which also suggests a change in structural flexibility.

The stability of G6PD variants was assessed upon treatment with trypsin in the absence and presence of different NADP^+^ concentrations (0, 10, and 100 μM). G6PD WT was the most stable protein, with or without NADP^+^, retaining the highest enzyme activity after trypsin treatment, followed in decreasing order by G6PD Kerala-Kalyan and G6PD Coimbra (Fig 2B).

To further evaluate the structural stability of G6PD variants, G6PD proteins were treated with increasing concentrations of Gdn-HCl (0 to 0.5 M). *C*_1/2_ was defined as the Gdn-HCl concentration at which the enzyme loses 50% of its activity, and the results are shown in Fig 2C. G6PD Coimbra and G6PD Kerala-Kalyan were structurally less stable than G6PD WT, with the *C*_1/2_ values of 0.26, 0.08, and 0.20 M for G6PD WT, G6PD Coimbra, and G6PD Kerala-Kalyan in the absence of NADP^+^, respectively. In the presence of NADP^+^, G6PD Coimbra still showed lower resistance to Gdn-HCl than that of G6PD WT and G6PD Kerala-Kalyan, indicating that the Coimbra mutation had a greater effect on structural stability than the Kerala-Kalyan mutation.

Fluorescence-based thermal shift assays were performed in the absence and presence of different NADP^+^ concentrations (0, 10, and 100 μM) to assess the structural stability of G6PD variants. Upon increasing the temperature, the protein structure unfolded, and the temperature at which half of the protein structure was unfolded was defined as *T*_*m*_, (Fig 2D). In the absence of NADP^+^, G6PD WT, G6PD Coimbra, and G6PD Kerala-Kalyan had *T*_*m*_ values of 52.31°C, 48.82°C, and 51.00°C, respectively. In agreement with the chemical denaturation analysis, G6PD Coimbra and G6PD Kerala-Kalyan were structurally less stable, showing 3.49°C and 1.31°C lower *T*_*m*_ values than that of the WT enzyme, respectively. The presence of NADP^+^ was found to improve structural stability of all proteins. The *T*_*m*_ values of 57.76°C, 54.84°C, and 55.43°C were observed for G6PD WT, G6PD Coimbra, and G6PD Kerala-Kalyan in the presence of 10 μM NADP^+^, respectively. Additionally, 100 μM NADP^+^ increased *T*_*m*_ values of G6PD WT, G6PD Coimbra, and G6PD Kerala-Kalyan by 8.55°C, 9.45°C, and 7.27°C, respectively, compared with no NADP^+^.

### Structural variants of *CYP2D6*

All samples were successfully amplified for the entire *CYP2D6* gene using DPKup and DPKlow primers (with expected size of 5.1 kb), except for one sample, which was found to have a complete loss of *CYP2D6* gene (S1 Fig). Deletion detection primers (5′2D6*5 and 3′2D6*5) amplified 12 samples while three samples had gene multiplication, with an expected size of 3.5 kb (S1 Fig).

For *CYP2D6* genotyping, samples positive for DPKup and DPKlow primers were subjected to nested PCR, which amplified three subregions (S2 Fig). Samples with *CYP2D6* gene multiplication were further confirmed by amplifying fragments C, D, and E (S3 Fig).

To identify the tandem CYP2D6*36+*10, 2D6-7S and 2D6-2AS primers were used to exclusively amplify the *CYP2D6* gene, and two nested primer sets (2D6E×7F6s-CYP32R and 2D7DownF1-2D62695R) were subsequently used (S4 Fig). Additionally, for *CYP2D6* gene duplication, 2D6E7F6s-CYP32R primers produced 4.8-kb PCR products.

### CYP2D6 alleles and phenotypes

*CYP2D6* gene DNA sequencing from 87 samples revealed a fairly high incidence of CYP2D6*10 in the studied population, with 36 samples carrying this genotype. Most *P*. *vivax*-infected patients carried the CYP2D6*36+*10 tandem type, accounting for 51.1%. Other detected genotypes included CYP2D6*1, *2, *4, *5, *20, *39, *41, *65, *69, *71, *72, *86, and *144. CYP2D6 allele and genotype frequencies in 88 *P*. *vivax*-infected patients identified in this study are shown in Tables 3 and 4.

**Table 3:** Frequency of *CYP2D6* allele and SNPs identified in this study.

Allele	Identified SNPs ^a^	Functional status	*N*	Frequency
*1	Reference allele	Normal	39	0.222
*2	G1662C, **C2851T**, **G4181C**	Normal	9	0.051
*4	C100T, C1038T, **G1847A**, G1662C, G4181C	No	5	0.028
	C100T, C973A, A983G, G1662C, **G1847A** (splice defect), G4181C			
	C100T, C973A, A983G, C996G, G1662C, **G1847A** (splice defect), G4181C			
	C973A, A983G, G1662C, **G1847A**			
*5	Deletion	No	15	0.085
*10	**C100T**, C1038T, G1662C, **G4181C**	Decreased	42	0.239
	**C100T**, C1038T, G1662C, **G4110A**, **G4181C**			
	**C100T**, C973A, A983G, C996G, G1662C, **G4181C**			
	**C100T**, C1038T, **G1916A**, G1662C, **G4181C**			
*20	G1662C,**1977_1978insG**, **C1979T+T1980C**, **C2851, G4181C**	No	1	0.006
*36+*10	**C100T**, C1038T, G1662C, **G4181C**, **Exon9Conv**	Decreased	45	0.256
	**C100T**, C1038T, G1662C, **G2583C**, **G4181C**, **Exon9Conv**			
*39	**G4181C**	Normal	4	0.023
	G1662C, **G4181C**			
*41	G1662C, **C2851T**, **G2989A** (splice defect), **G4181C**	Decreased	7	0.040
*65	**C100T**, C1038T, G1662C, **C2851T**, **G4181C**	Uncertain	2	0.011
*69	**C100T**, C1038T, G1662C, **C2851T**, **G2989A** (splice defect), **G4181C**	No	2	0.011
*71	**G125A**	Uncertain	1	0.006
*72	**C100T**, **G3319A**, **G4181C**	Uncertain	1	0.006
*86	**G2607A**, **T2611A**	Unknown	1	0.006
*144	**G2441A** (splice defect)	No	2	0.011
**Total**			**176**	**1**

^a^ Key SNPs of each allele are bolded. Novel mutations are bolded and underlined.

**Table 4:** Frequency of genotypes and predicted phenotype of CYP2D6.

Genotype	Activity score ^a^	Predicted phenotype ^b^	*N*	Frequency
*1/*1	2	NM	8	0.091
*1/*2	2	NM	1	0.011
*1/*41	1.5	NM	1	0.011
*1/*10	1.25	NM	8	0.091
*1/*36+*10	1.25	NM	10	0.114
*2/*36+*10	1.25	NM	7	0.080
*36+*10/*39	1.25	NM	1	0.011
*36+*10/*69	1.25	NM	1	0.011
*1/*144	1	IM	1	0.011
*1/*5	1	IM	2	0.023
*2/*5	1	IM	1	0.011
*39/*69	1	IM	1	0.011
*10/*41	0.75	IM	3	0.034
*36+*10/*41	0.75	IM	2	0.023
*4/*41	0.5	IM	1	0.011
*10/*10	0.5	IM	6	0.068
*10/*36+*10	0.5	IM	14	0.159
*4/*10	0.25	IM	2	0.023
*4/*36+*10	0.25	IM	1	0.011
*5/*10	0.25	IM	2	0.023
*5/*36+*10	0.25	IM	8	0.091
*20/*36+*10	0.25	IM	1	0.011
*5/*5	0	PM	1	0.011
*4/*72	n/a	Indeterminate	1	0.011
*10/*71	n/a	Indeterminate	1	0.011
*39/*65	n/a	Indeterminate	2	0.023
*86/*144	n/a	Indeterminate	1	0.011
**Total**			**88**	**1**

^a^ n/a, no value assigned for AS calculation.

^b^ PM, poor metabolizer; IM, intermediate metabolizer; NM, normal metabolizer.

DNA sequencing also revealed three novel mutations, G1916A, G2583C, and G4110A, which are located in exons 4, 5, and 9, respectively. The G1916A and G2583C mutations resulted in the amino acid changes Gly192Arg and Asp270His, respectively, while G4110A was a silent mutation. The effects of Gly192Arg and Asp270His mutations on the catalytic activity of the CYP2D6 enzyme requires further investigation.

## Discussion

Primaquine and tafenoquine are antimalarial drugs that can prevent relapse by killing dormant *P*. *vivax* and *P*. *ovale* liver stages. Furthermore, primaquine is recommended as an essential co-drug for killing *P*. *falciparum* gametocytes and thus preventing malaria transmission from humans to mosquitoes [5]. Because of their unique and broad spectrum of antimalarial activity, primaquine and tafenoquine are tools that can support malaria elimination. However, these 8-aminoquinolines are often underused because both primaquine and tafenoquine can cause a dose-dependent hemolytic toxicity in G6PD-deficient individuals. Thus, before prescribing 8-aminoquinolines, G6PD activity must be tested to assess the hemolytic risk. G6PD-deficient individuals with <30% activity should not be treated with a primaquine standard dose (15 mg/day for 14 days) but should instead be given 0.75 mg/kg weekly (45 mg/week) for 8 weeks under close medical supervision [11]. Tafenoquine should only be administered to patients with G6PD activity greater than 70% because it cannot be stopped if adverse hemolysis is detected [7]. However, G6PD screening is not routinely performed, particularly in small malaria clinics along Thailand’s borders.

**Fig 3:**
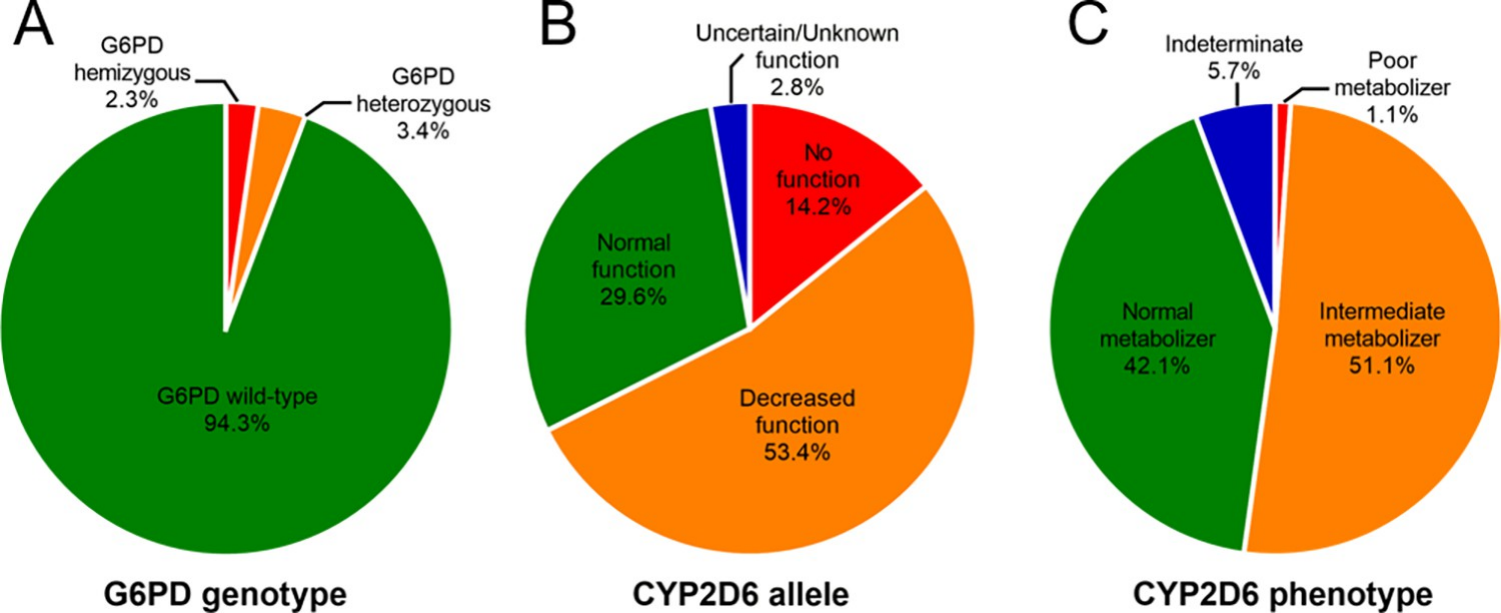
Profile of G6PD deficiency and CYP2D6 in the studied population. The distribution of (A) G6PD genotypic status (n = 88), (B) *CYP2D6* functional alleles (n = 176), and (C) predicted CYP2D6 phenotype (n = 88).

Among vivax patients in Yala province, multiplexed HRM assays identified a G6PD-deficient male and heterozygous female as having G6PD Kaiping and G6PD Mahidol, respectively. DNA sequencing confirmed the presence of known G6PD variants in three cases, including G6PD Vanua Lava, G6PD Coimbra, and G6PD Kerala-Kalyan (Fig 3A). Four other cases were found to have C1311T and IVS XI T93C polymorphisms, which are common in G6PD-deficient Asians [42–44].

G6PD Kaiping, G6PD Vanua Lava, and G6PD Coimbra were Class B variants and potentially susceptible to 8-aminoquinoline hemolytic toxicity [45,46]. G6PD Mahidol was previously reported to be the most common variant in southern Thailand, and it is also commonly found in Malaysia [29,31,47,48]. However, G6PD Viangchan and G6PD Mediterranean (Class B variants), the other two most common variants among Malays, were not found in our study [47,48]. G6PD Mahidol showed a wide range of enzyme activities, with some women exhibiting G6PD activity greater than the 70% of cut-off; however, acute hemolytic anemia was reported in G6PD Mahidol heterozygous women with normal phenotypes when they were taking 8-aminoquinolines [49]. G6PD Kerala-Kalyan was common in India, but it was first reported outside of India in Phuket, Thailand in 2006 [31]. Our study is the second report on this variant in Thailand, indicating the probability of G6PD gene flow from India to Thailand.

**Fig 4:**
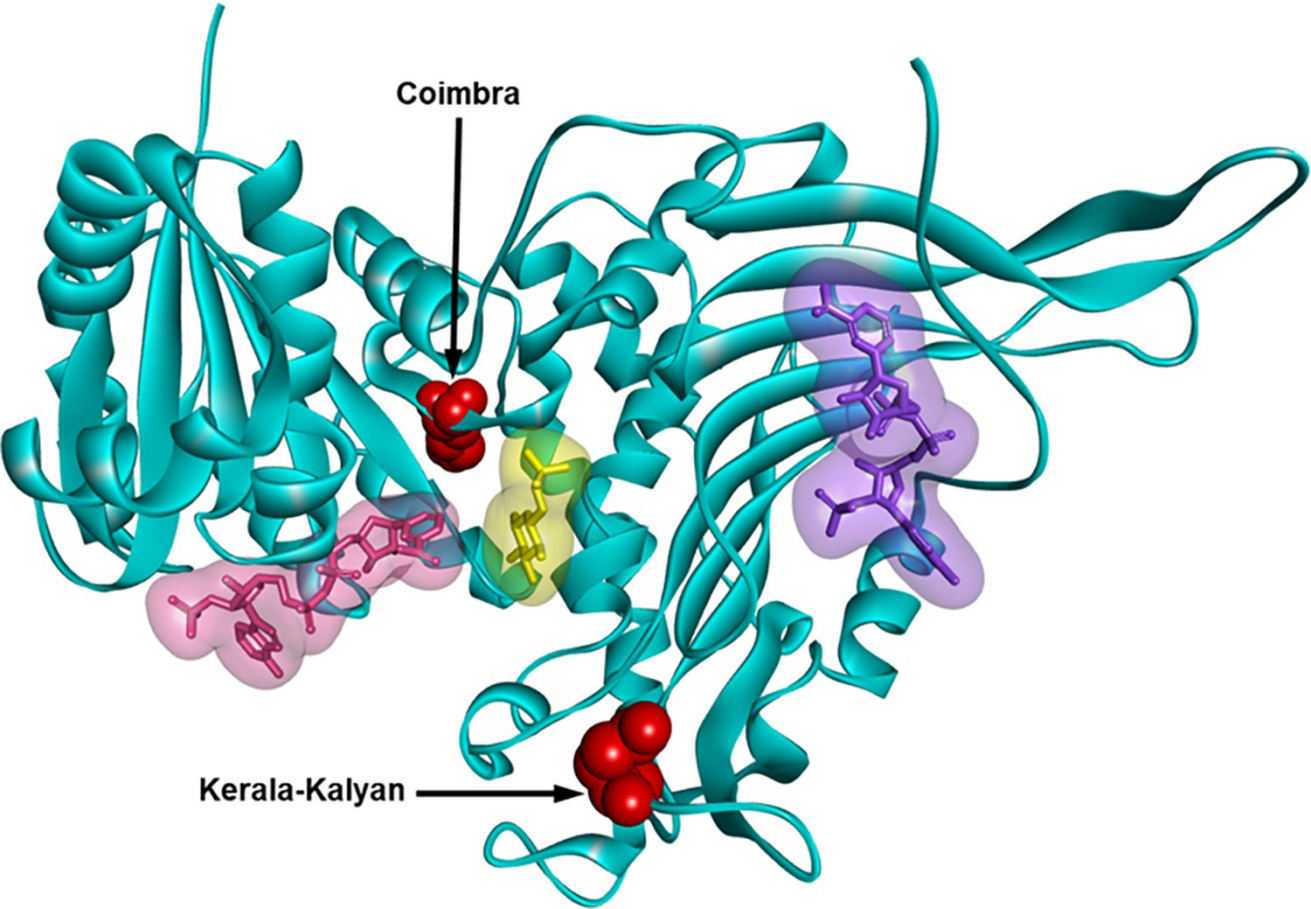
Three-dimensional structure of human G6PD enzyme (PDB: 2BHL and 2BH9). The structural NADP^+^, NADP^+^ coenzyme, and G6P substrate are shown as purple, pink, and yellow molecular surface representations, respectively. The mutants are shown in CPK representation. The graphical representation was constructed using Discovery Studio Visualizer-Accelrys.

In this study, we provided insight into the biochemical properties of G6PD Coimbra and G6PD Kerala-Kalyan by determining the catalytic activity and structural stability of these two variants. G6PD Coimbra markedly reduced catalytic activity by 11-fold compared with the WT enzyme. The Coimbra mutation increased the binding affinity for G6P without altering binding towards the NADP^+^ coenzyme, which was consistent with previous findings [50]. On the basis of the three-dimensional structure, the Coimbra mutation (Arg198Cys) is located in the conserved region (residues 198–206), which is responsible for substrate binding and catalysis (Fig 4) [39]. Moreover, the Coimbra mutation was found to reduce protein structural stability, as evidenced by decreases in thermal denaturation and susceptibility to Gdn-HCl and trypsin treatment. Individuals with G6PD Coimbra are thus considered susceptible to hemolysis triggers, particularly those with homozygous and hemizygous genotypes who may experience acute hemolytic anemia after 8-aminoquinoline administration if their G6PD activity is sufficiently low.

The Glu317Lys mutation in the G6PD Kerala-Kalyan is located far from the structural NADP^+^, catalytic NADP^+^, and G6P binding sites (Fig 4), and our findings revealed that the mutation had only a minor effect on catalytic activity and protein stability. These results suggest that the Kerala-Kalyan mutation may partially contribute to the mild to severe drug-induced hemolysis. Our suggestion is in agreement with the clinical data reported in a previous study. An individual with G6PD Kerala-Kalyan who had a G6PD activity of 45% experienced mild hemolysis after receiving 0.75 mg/kg/week of primaquine [51]. Although our study provides detailed information about the activity and stability of G6PD variants, it should be noted that hemolytic risk in an individual is influenced by a variety of factors and cannot be predicted solely by biochemical analysis.

While G6PD deficiency was discovered in approximately 8% of people living in malaria-endemic areas, impaired CYP2D6 activity was estimated to affect more than 20% of the population [10,52]. Our findings showed that the frequencies of the null, decreased, and normal function alleles were 14.2%, 53.4%, and 29.6%, respectively (Fig 3B). The increased activity alleles were not found in the current study. Among the 15 variants found in the studied population, the five most common alleles were CYP2D*36+*10 (25.6%), *10 (23.9%), *1 (22.2%), *5 (8.5%), and *2 (5.1%). The frequency of CYP2D6*10 was lower than that in previous reports in Thais (approximately 50%) [34,36,53]. It was suggested that the CYP2D6*10 frequency was overestimated in the past because the available platforms were unable to detect structural variants. Luminex, MassArray, and TaqMan platforms cannot detect the *CYP2D6-2D7* hybrid of *36, leading to an erroneous assignment of CYP2D*36+*10 as *10 [34,54]. In the current study, we performed long-range PCR with a *36-specific primer set described by Soyama *et al*. to reliably detect the tandem structure [55]. A comprehensive analysis of CYP2D6 alleles among Asians living in Hong Kong revealed similar results as those in our study. The five most common alleles were CYP2D6*36+*10 (34.1%), *1 (23.1%), *10 (21.6%), *2 (9.7%), and *5 (3.0%) [56]. Furthermore, the CYP2D6*36+*10 allele was found with a frequency range of 24% to 32% in the Japanese population [55,57,58]. Thus, our study provided a novel finding about the CYP2D6*36+*10 frequency in Thailand and suggested that this tandem arrangement was a major allele among Asians.

Both CYP2D6*10 and *36+*10 are decreased-function alleles. An *in vitro* study revealed that *10 markedly reduced CYP2D6 activity by 60% to 90% for several drugs [59–61]. Additionally, no primaquine 5-hydroxylation activity was detected for CYP2D6*10, suggesting the contribution of this allelic variant to the therapeutic failure of primaquine [62]. The effect of CYP2D6*36+*10 on drug metabolism has not been fully characterized, but this tandem variant seems to be functionally equivalent to *10 alone [57]. CYP2D6*5 was a gene deletion, resulting in no enzyme activity. Two novel missense mutations, G1916A and G2583C, were found in our samples carrying CYP2D6*10 and *36+*10, respectively. The G1916A mutation causes an amino acid change at position 192 from glycine to arginine, while G2583C results in the conversion of aspartic acid at 270 to histidine. G1916A and G2583C mutations should be studied further because they are located within coding regions and are likely to affect CY2D6 activity (S5 Fig).

In the present study, 27 CYP2D6 allele combinations were observed. CYP2D6*10/*36+*10 was the predominant genotype (15.9%), followed by CYP2D6*1/*36+*10 (11.4%). Five diplotypes, CYP2D6*1/*1, *1/*10, *2/*36+*10, *5/*36+*10, and *10/*10, were detected at frequencies of 7% to 9%. Because the tandem diplotype of *36+*10 is often undetected, there are limited *in vivo* data on genotypes carrying CYP2D6*36+*10. However, an individual with CYP2D6*10/*36+*10 and *5/*36+*10 can be referred to as *10/*10 and *5/*10, respectively. CYP2D6*10/*10, which is predicted to be an intermediate metabolizer, negatively affected the treatment outcome for *P*. *vivax*. A patient with this diplotype had three episodes of *P*. *vivax* infection within 3 months after completing a high-dose primaquine course of therapy (0.5 mg/kg for 14 days) [63]. Furthermore, a clinical trial reported that 52.9% of patients harboring CYP2D6*10/*10 experienced relapse within 1 year after high-dose primaquine therapy [18]. Additionally, 62.5% of vivax patients carrying the *5/*10 genotype were reported to experience malaria relapse [18]. It is assumed that 50% of people with an intermediate phenotype are at risk of developing primaquine failure. Among the patients in this study, 51.1% were presumed intermediate metabolizers, suggesting that approximately 25% of vivax patients in the area may have a poor response to primaquine, and thus, may be at risk of relapse (Fig 3C).

In 88-vivax patients from Yala province, the mutation frequency was 5.68% and Class B G6PD variants with a risk of mild to severe drug-induced hemolysis were identified. A previous study with a larger sample size reported G6PD deficiency in 1.1% (6/562) of the 562 malaria patients in Yala province [29]. Using DiaplexC G6PD genotyping kit (Asian type), they identified G6PD Mahidol and G6PD Viangchan with the frequency of 0.5% and 0.4% in vivax malaria patients (n = 545). Another study in healthy volunteers revealed that the prevalence of G6PD deficiency in southern Thailand was approximately 8% (42/520) and Yala province has G6PD deficiency of 3.33% (1/30) [64]. Thus, our results suggest that screening for G6PD deficiency prior to 8-aminoquinoline therapy is necessary in this region to avoid unexpected outcomes. There are several point-of-care diagnostics for G6PD deficiency that can be extended to international borders and small malaria clinics, which could aid in addressing the problem of G6PD deficiency in the area. For CYP2D6, our findings revealed that approximately 51.1% of the vivax patients studied were predicted to be intermediate metabolizers, and null and decreased functional alleles were observed with a high frequency (Fig 3B and 3C). Thus, the number of allele combinations resulting in poor and intermediate genotypes is expected to increase in the future. In contrast to G6PD testing, genetic testing of CYP2D6 is currently limited in clinical use. Thus, our research adds to the growing concern about the use of primaquine in this area. To support the malaria elimination program, a policy to ensure the safety and effectiveness of a radical cure should be established.

The limitations of the current study should be noted. Here, we chose to study on only individuals infected with vivax who actually face the problem of 8-aminoquinoline use. Thus, each G6PD variant (G6PD Vanua Lava, G6PD Coimbra, G6PD Mahidol, G6PD Kerala-Kalyan, and G6PD Kaiping) was detected in only one sample due to the small sample size (88 samples), which is approximately 4% of all vivax cases reported in Yala province. Additional research with a larger population is recommended to confirm the findings of our study. Because it was hypothesized that some G6PD mutations confer a protective effect against malaria infection, some G6PD variants with reduced susceptibility to malaria infection may have been excluded from our study and the results were not representative prevalence of whole population in Yala province. Despite the fact that the CYP2D6 phenotype was not directly measured in this study, genetic testing is a current and acceptable tool for determining the functional status of the CYP2D6 enzyme. Finally, there are no clinical data analyzed here; a future comprehensive study including such information would provide a better understanding of the implementation of G6PD and CYP2D6 testing on the use of 8-aminoquinolines for vivax malaria elimination.

## Supporting information

S1 Table Primers used for site-directed mutagenesis. (XLSX)

S2 Table Primers used for CYP2D6 amplification and genotyping. (XLSX)

S1 Fig Agarose gel electrophoresis of CYP2D6 gene amplification. Primers: DPKup and DPKlow, 5′2D6*5 and 3′2D6*5 and 2D6dupl-F and 2D6dupl-R were used to amplify the entire CYP2D6, detect the CYP2D6 deletion and multiplication, respectively. M, 1 kb DNA marker and lanes 1–6 indicated sample numbers.
(TIF)

S2 Fig Agarose gel electrophoresis of nested PCR of *CYP2D6* gene. The 1^st^ PCR products were used as a template to amplify 3 subregions. The expected sizes of amplicons 1, 2 and 3 were 1,350 bp, 1,110 bp and 1,652 bp, respectively. M, 1 kb DNA marker and lanes 1–17 indicated sample numbers. The PCR products were purified and sequenced.
(TIF)

S3 Fig Agarose gel electrophoresis of fragments C, D and E. The expected sizes of fragments C, D and E were 9.3 kb, 8.6 kb and 4.9 kb, respectively. M, 1 kb DNA marker and lanes 1–3 indicated sample numbers.
(TIF)

S4 Fig Agarose gel electrophoresis of nested PCR of *CYP2D6* gene for detecting tandem structure. The 1^st^ PCR products from the 2D6-7S and 2D6-2AS primers were used as a template to amplify 2 subregions. The expected sizes of amplicons generated by the primers 2D6E7F6s-CYP32R and 2D7DownF1-2D62695R were 6.4 and 5.5 kb, respectively. M, 1 kb DNA marker and lanes 1–18 indicated sample numbers. Sample 8 generated a 4.8 kb product by the primers 2D6E7F6s-CYP32R, indicating *CYP2D6* gene duplication.
(TIF)

S5 Fig Three-dimensional structure of human CYP2D6 enzyme (PDB: 3TBG). The heme prosthetic group and thioridazine substrate are shown as pink and yellow molecular surface representations, respectively. The mutants are shown in CPK representation. The graphical representation was constructed using Discovery Studio Visualizer-Accelrys.
(TIF)
