# Supplementary figures and images for: Cytochrome P450 2D6 (CYP2D6) and glucose-6-phosphate dehydrogenase (G6PD) genetic variations in Thai vivax malaria patients: Implications for 8-aminoquinoline radical cure
Source: PLoS Negl Trop Dis. 2022 Dec 12;16(12):e0010986. doi: 10.1371/journal.pntd.0010986 (PMC9779666; doi:10.1371/journal.pntd.0010986)

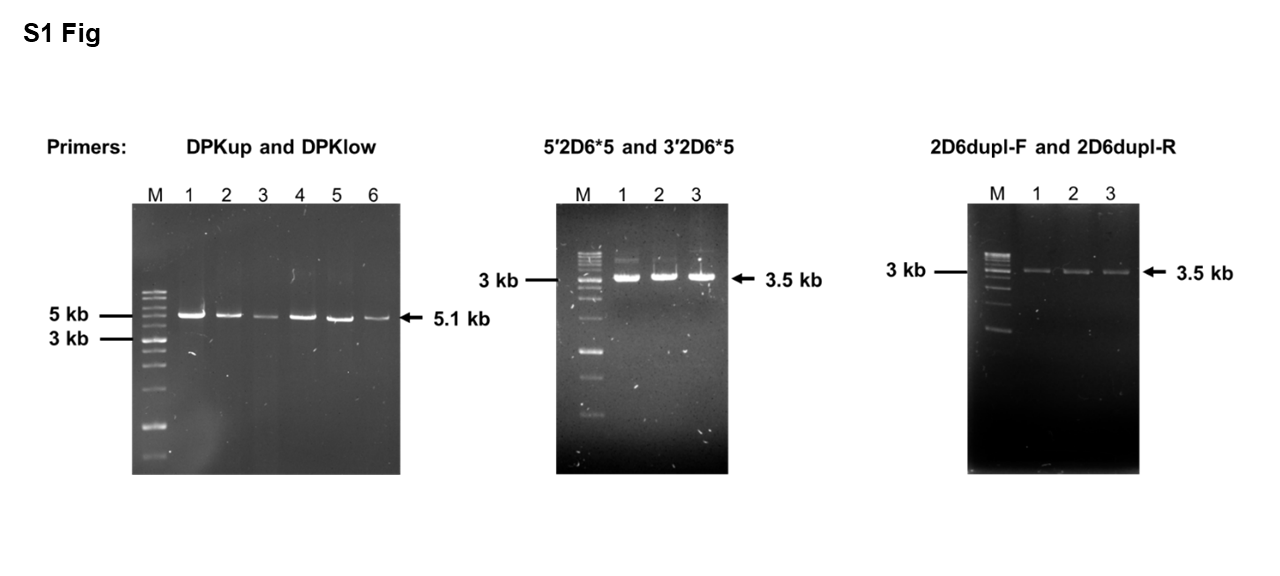

Supplement: S1 Fig — Primers: DPKup and DPKlow, 5′2D6*5 and 3′2D6*5 and 2D6dupl-F and 2D6dupl-R were used to amplify the entire CYP2D6, detect the CYP2D6 deletion and multiplication, respectively. M, 1 kb DNA marker and lanes 1–6 indicated sample numbers. (TIF) [file pntd.0010986.s003.tif]

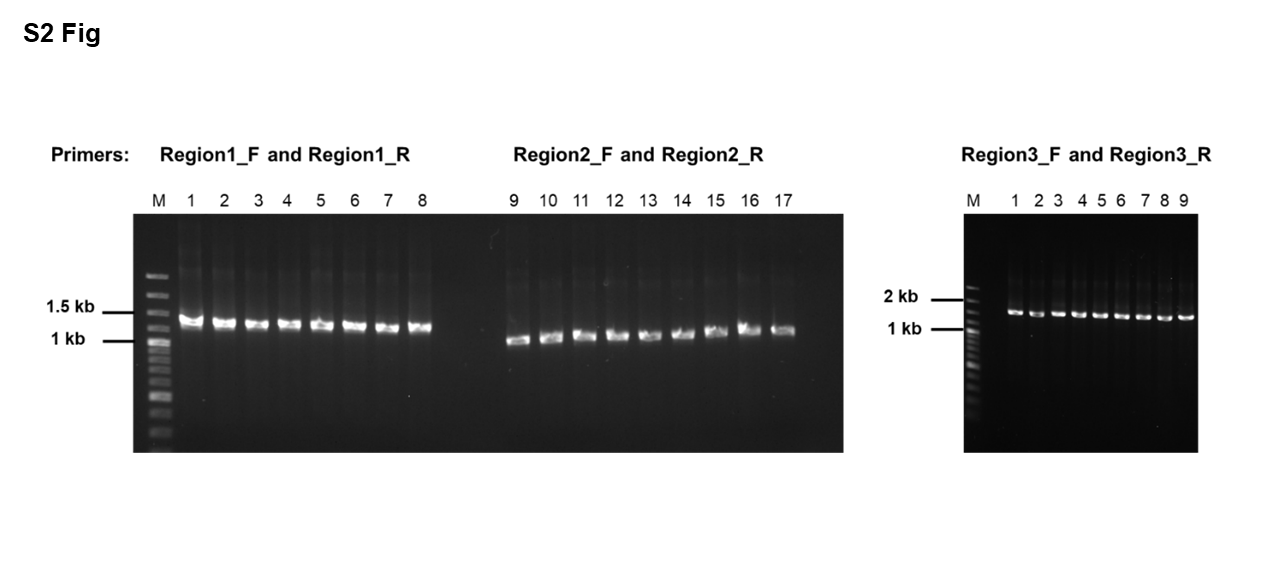

Supplement: S2 Fig — The 1st PCR products were used as a template to amplify 3 subregions. The expected sizes of amplicons 1, 2 and 3 were 1,350 bp, 1,110 bp and 1,652 bp, respectively. M, 1 kb DNA marker and lanes 1–17 indicated sample numbers. The PCR products were purified and sequenced. (TIF) [file pntd.0010986.s004.tif]

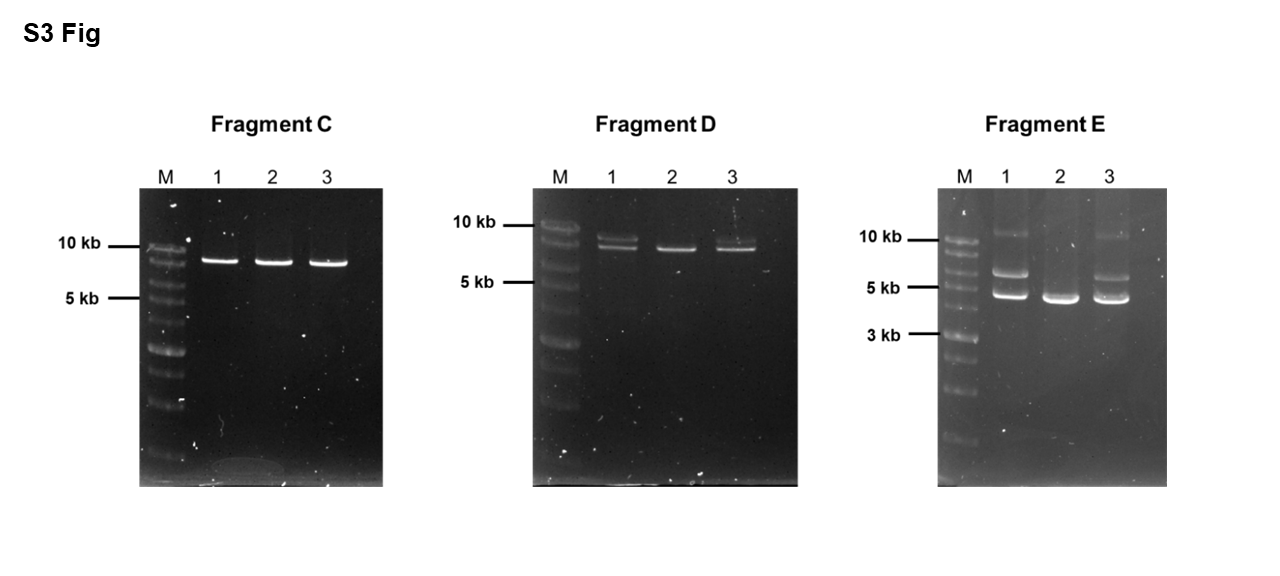

Supplement: S3 Fig — The expected sizes of fragments C, D and E were 9.3 kb, 8.6 kb and 4.9 kb, respectively. M, 1 kb DNA marker and lanes 1–3 indicated sample numbers. (TIF) [file pntd.0010986.s005.tif]

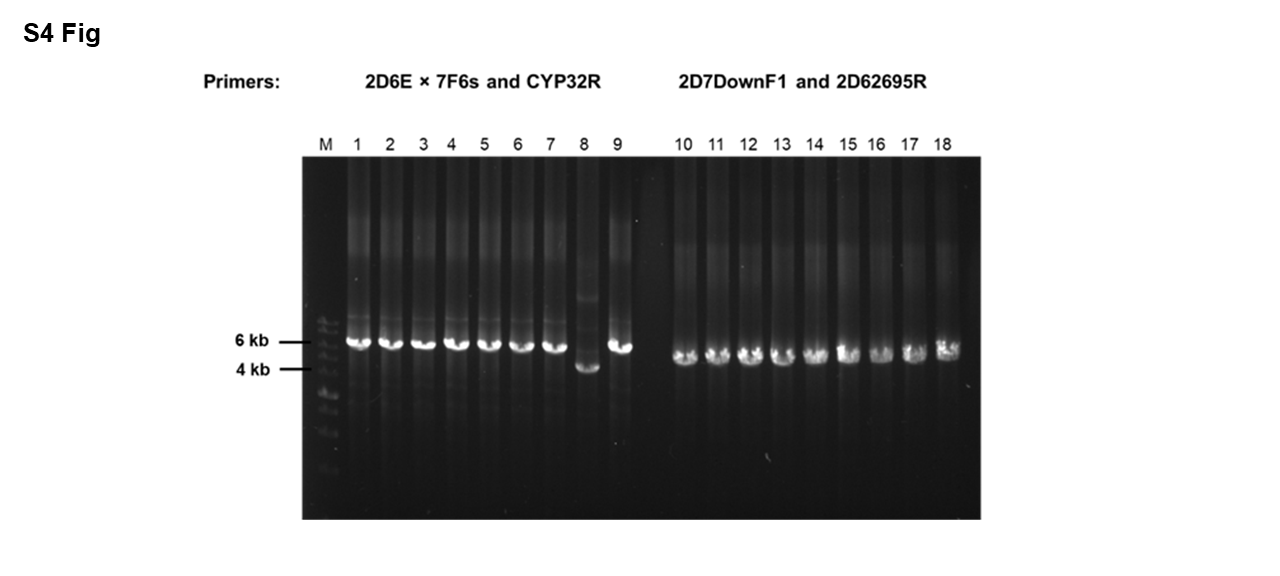

Supplement: S4 Fig — The 1st PCR products from the 2D6-7S and 2D6-2AS primers were used as a template to amplify 2 subregions. The expected sizes of amplicons generated by the primers 2D6E7F6s-CYP32R and 2D7DownF1-2D62695R were 6.4 and 5.5 kb, respectively. M, 1 kb DNA marker and lanes 1–18 indicated sample numbers. Sample 8 generated a 4.8 kb product by the primers 2D6E7F6s-CYP32R, indicating CYP2D6 gene duplication. (TIF) [file pntd.0010986.s006.tif]

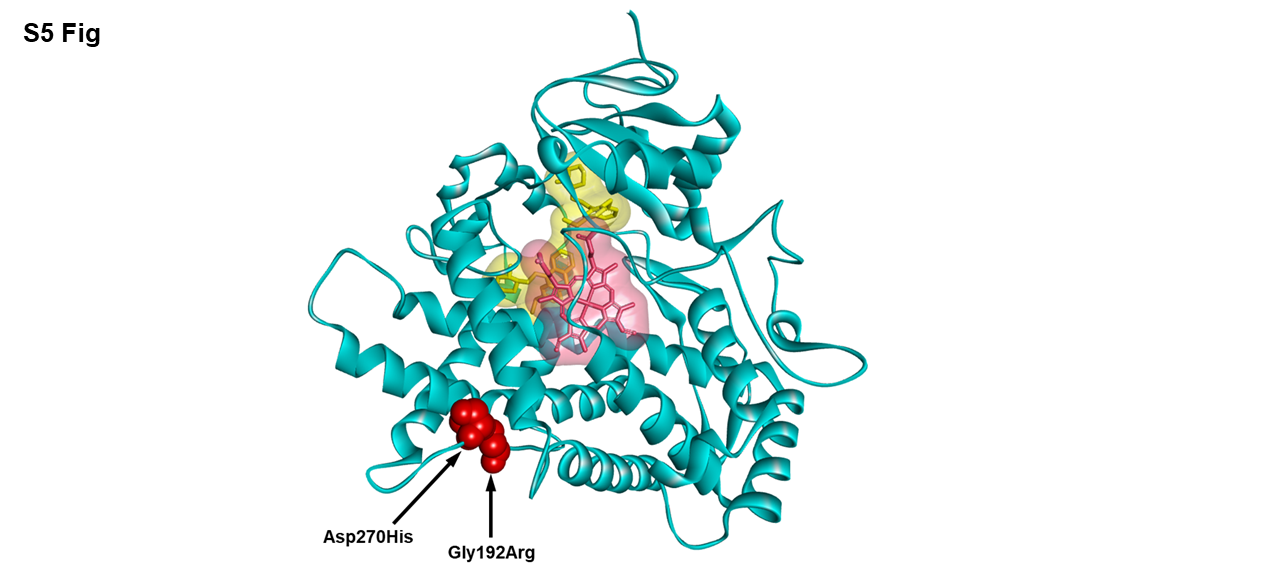

Supplement: S5 Fig — The heme prosthetic group and thioridazine substrate are shown as pink and yellow molecular surface representations, respectively. The mutants are shown in CPK representation. The graphical representation was constructed using Discovery Studio Visualizer-Accelrys. (TIF) [file pntd.0010986.s007.tif]
